# Object-Based Integration of Photogrammetric and LiDAR Data for Automated Generation of Complex Polyhedral Building Models

**DOI:** 10.3390/s90705679

**Published:** 2009-07-15

**Authors:** Changjae Kim, Ayman Habib

**Affiliations:** Department of Geomatics Engineering, The University of Calgary, 2500 University Drive NW, T2N 1N4, Calgary, Canada; E-Mail: cjkim@ucalgary.ca

**Keywords:** polyhedral building model generation, complex structures, data integration, LiDAR, photogrammetry, hierarchical processing

## Abstract

This research is concerned with a methodology for automated generation of polyhedral building models for complex structures, whose rooftops are bounded by straight lines. The process starts by utilizing LiDAR data for building hypothesis generation and derivation of individual planar patches constituting building rooftops. Initial boundaries of these patches are then refined through the integration of LiDAR and photogrammetric data and hierarchical processing of the planar patches. Building models for complex structures are finally produced using the refined boundaries. The performance of the developed methodology is evaluated through qualitative and quantitative analysis of the generated building models from real data.

## 1. Introduction

Nowadays, there are increasing demands for up-to-date three-dimensional building models in various applications such as urban design and planning, 3D city modeling, disaster management, real-estate industry, and military training. Hence, the generation of a Digital Building Model (DBM) has been one of the key research topics in both the photogrammetric and computer vision communities. Significant research related to this topic has been implemented for the last two decades. Aerial imagery has traditionally been one of the preferred data sources for DBM generation, where a variety of approaches have been investigated and developed using either single image, stereo-image pair, or multiple images. The approaches utilizing single images are only effective for simple buildings [1,2]. Thus, much more research has been focused on the utilization of stereo and multiple images to acquire more reliable results [3–8]. Although satisfactory results are achieved in some cases, feature matching in large scale imagery over urban areas still remains to be an ill-posed problem since there are significant relief displacements, scale differences, geometric distortions, and occlusions in the imagery. Therefore, a low degree of automation in DBM generation from imagery has been reported in earlier research [9].

LiDAR (Light Detection and Ranging) systems, on the other hand, directly provide accurate and highly dense surface information without the need for feature matching in overlapping strips. Thus, several researchers have been interested in the utilization of LiDAR data for DBM generation [10–14]. In such approaches, the boundaries of the generated DBM are of lower quality due to the irregular and sparse nature of LiDAR data. Hence, the accuracy of the generated building boundaries is highly dependent on the LiDAR point density.

It is well known that image and LiDAR data have complementary characteristics, which would lead to a higher level of automation and more reliable DBM generation [15]. Therefore, several researchers have focused on the integration of LiDAR data and aerial imagery to acquire a higher level of automation and more reliable DBM [9,16–20]. For example, satisfactory results are demonstrated by [17,18], where LiDAR data was utilized to detect building regions and to reconstruct initial 3D building models. The initial building models are then refined by integrating LiDAR data with imagery. However, these approaches are limited to buildings with either simple or pre-defined shapes. A split-merge-shape technique using 3D edges derived from the integration of LiDAR data and a single aerial image was utilized by [19]. This research might have a significant problem in the process of generating 3D line segments since linear features derived from only a single image are utilized in the process. In other words, other linear features which are closely located to the actual building boundaries (i.e., linear features on the walls or roads) might be chosen to represent the building boundary segments. In [20], coarse building boundaries from LiDAR data with the assistance of color segmentation in a single aerial image were derived. Then, precise boundaries were defined by replacing the coarse boundaries with line segments from the image. This research might have the same problem which is caused by utilizing linear features extracted from a single image as mentioned above. Moreover, the quality of the results strongly depends on successful derivation of the coarse building boundaries. Some of the previous work were limited to simple buildings with either regular or pre-defined shapes. Moreover, other approaches have utilized linear features derived from only a single image without taking the advantage of available stereo-imagery and the incorporated LiDAR data as a constraint.

**Figure 1.**
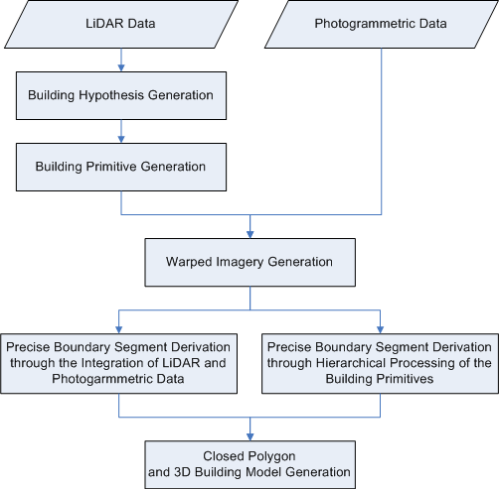
A flow diagram of the proposed DBM generation procedure.

This paper proposes a new methodology for automated DBM generation while overcoming the problems in the previous research. Complex building structures, whose rooftops can be represented by several planar patches bounded by straight lines, will be the main focus in this research. The planar patches might have different slopes and aspects. Since it is hard to represent complex building structures by pre-defined building models or building parts, this research will reconstruct polyhedral models through a data-driven approach. To achieve reliable building models for complex structures, this research focuses on the solutions of several problems. More specifically, geometric and spectral constraints are introduced to determine precise boundary segments. Moreover, a solution for the occlusion problem in large scale imagery over urban environment is suggested through hierarchical processing of building primitives (i.e., the individual planar patches constituting building rooftops). A flow diagram of the proposed DBM generation procedure is depicted in Figure 1. This paper starts by manipulating LiDAR data for building hypothesis generation and derivation of initial boundaries of the building primitives. Afterwards, the initial boundaries are refined through the integration of LiDAR and photogrammetric data and hierarchical processing of the building primitives. Building models for complex structures are then produced using the determined precise boundaries. The experimental results section presents the results from real data together with qualitative and quantitative evaluations of the derived DBM. Finally, conclusions and recommendations for future work are summarized.

## 2. Building Hypothesis and Primitive Generation from LiDAR Data

**Figure 2.**
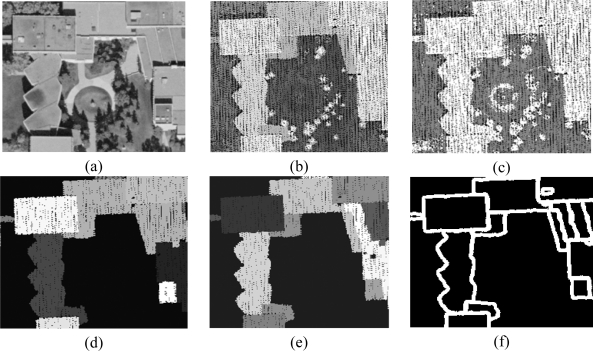
A dataset with complex and connected structures: (a) aerial photo; (b) LiDAR data; (c) classified terrain and off-terrain points; (d) generated building hypotheses; (e) building primitives; and (f) initial boundaries of the building primitives.

The proposed method starts with building hypothesis generation (i.e., building detection) which differentiates buildings from other objects (i.e., terrain, trees, cars, etc.) within the dataset. Since elevation data is directly acquired by a LiDAR system, the degree of automation in building detection using this type of data is higher when compared to that using imagery [9]. Hence, building detection is implemented through the manipulation of LiDAR data only. First, the classification of LiDAR data into terrain and off-terrain points is conducted through the identification of the occluding points (i.e., the points causing the occlusion), which are hypothesized to be off-terrain points [21,22]. Once the LiDAR point cloud has been separated into terrain and off-terrain points, the identified off-terrain points are further classified into points belonging to planar surfaces and to rough surfaces through an iterative plane fitting and roughness test. For more details regarding this procedure, one can refer to [23]. Afterwards, the points belonging to planar surfaces are grouped together according to their three-dimensional proximity. The area and the height of the resulting groups relative to neighbouring terrain points will be used for building hypothesis generation with the help of thresholds that define the minimum area and height of the buildings in the study area. Figure 2a–d shows an example of an aerial image patch, corresponding LiDAR data, classified terrain and off-terrain points, and building hypotheses, respectively. The different shades of grey in Figure 2(d) indicate different building hypotheses.

A generated building hypothesis might be composed of several connected planar patches. Therefore, the segmentation procedure suggested by [14] is carried out to break each building hypothesis down into a group of building primitives (i.e., planar patches constituting rooftops). The segmentation procedure is based on a voting scheme that keeps track of the point attributes, as defined by a local plane through its neighbouring points, in an accumulator array. While globally assessing the frequency of the local attributes in the parameter space together with the proximity of the points in the object space at the same time, the points belonging to different planar patches are reliably identified and grouped into different clusters. As a next step, the minimum convex hull procedure [24] is used to define the initial boundaries of the derived building primitives. Figure 2e,f illustrates the generated building primitives and their boundaries, respectively. As shown in Figure 2(f), the derived initial boundaries represent the trends of the building shapes well. However, the quality of the derived boundaries is not high enough to be utilized for precise building model generation due to the irregular and sparse nature of LiDAR data.

**Figure 3.**
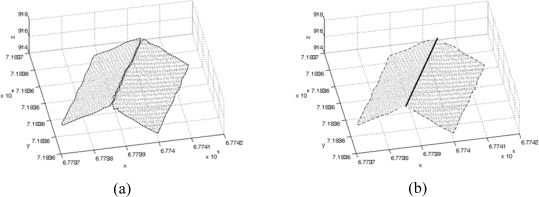
(a) Initial boundaries of the primitives and (b) the precise boundary segment through the intersection of two neighbouring primitives and the remaining initial boundary points.

Hence, there is need for the improvement of the initial boundaries of the building primitives to acquire a precise building model. Since the building primitives produced have accurate plane parameters, a precise boundary segment for a ridge line can be generated by intersecting two adjacent building primitives if any exist. The intersection of the building primitives that are almost parallel and/or coplanar is avoided by checking the angles between the surface normal vectors of the primitives since this would lead to a weak intersection. Figure 3(a) shows a real example of the initial boundaries of the primitives (i.e., dashed lines). Once a boundary segment has been determined through the intersection process, all the initial boundary points that are closely located to the segment are identified and removed from the boundary in question. Figure 3(b) shows a boundary segment (i.e., solid line) derived through the intersection of neighbouring building primitives and the remaining boundary points. Only the remaining boundary points will be utilized in the following boundary refinement processes in Section 3 and 4. It is worth noting that the refinement processes will handle one building primitive at a time.

## 3. Precise Boundary Segment Derivation through the Integration of LiDAR and Photogrammetric Data

Precise boundary segments for the remaining portions of the building boundaries will be derived through the integration of LiDAR data and stereo imagery. This procedure will be carried out through several steps: 1) Co-registration of LiDAR and image data; 2) Warped image generation; 3) Straight line detection; 4) 3D line matching; 5) Grouping of matched 3D lines; and 6) Precise boundary segment selection. These steps will be explained in the following paragraphs.

### 3.1. Co-registration of LiDAR and Image Data

As a prerequisite of the integration procedure, the co-registration of the imagery and LiDAR data to a common reference frame is carried out. To ensure the best quality of the co-registration procedure, the LiDAR data is utilized as the source of control for the image geo-referencing. Since it is almost impossible to identify distinct LiDAR points, which can be recognized in the imagery, segmented planar patches and extracted linear features from the LiDAR data are utilized as the source of control for the geo-referencing of the aerial imagery [25].

### 3.2. Warped Imagery Generation

It is well know that large scale imagery over urban areas has significant relief displacement and geometric distortions which are factors that make image matching difficult and unreliable. To resolve this problem, we decided to incorporate LiDAR data into the matching procedure as a constraint. Let us assume at this stage that two orthophotos are generated by using a stereo-image pair and LiDAR data (i.e., Digital Surface Model). Since the ortho-rectification procedure eliminates perspective distortions, features in one orthophoto will be directly compatible with their conjugate features in the other orthophoto. Hence, the feature matching process using these orthophotos will be much easier and more reliable compared to the process using the original imagery. However, this research will not generate a complete orthophoto for each of the images of a stereo-pair. Instead, we will generate pseudo-orthophotos that only rectify a portion of the image that includes the building primitive under consideration. These pseudo orthophotos will be denoted as “warped imagery”. In this case, the agreement of the features in the warped imagery will exist only along the rooftop patch.

**Figure 4.**
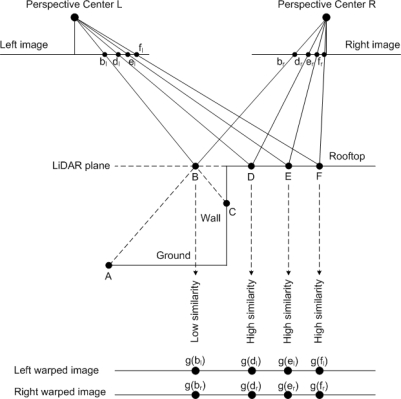
Concept of warped imagery.

Two warped images are generated through the projection of the stereo-image pair onto the plane of the relevant building primitive generated from the LiDAR data. More specifically, the building primitive is extended along the building primitive plane (in the object space) beyond the derived initial boundary while considering the resolution of the LiDAR data. Object points, which are regularly spaced according to the nominal Ground Sampling Distance (GSD) of the aerial images, within the range of the building primitive plane in question are then projected onto the corresponding image plane using the collinearity equations and the established geo-referencing parameters. For example, the object points, B, D, E, and F along the LiDAR plane are projected onto the corresponding image points in the left image (i.e., b_l_, d_l_, e_l_, and f_l_); see Figure 4. In the same manner, these object points are projected onto the corresponding image points in the right image (i.e., b_r_, d_r_, e_r_, and f_r_). Then, the grey value at the projected image location (e.g., a grey value, g(d_l_) at the position, d_l_ in the left image) is assigned to the corresponding location of the object point (e.g., point D) on the building primitive plane (or LiDAR plane in Figure 4).

As can be seen in the figure, the degree of similarity between the two warped images (i.e., left and right warped images at the bottom of the figure) for a location on the rooftop of a building primitive will be high since the corresponding points in the left and right images are conjugate; refer to points D, E and F and their grey values on the warped images (g(d_l_) & g(d_r_), g(e_l_) & g(e_r_), and g(f_l_) & g(f_r_)). The degree of similarity for a point that does not physically belong to the building rooftop (e.g., point B in Figure 4), on the other hand, will be lower since the projected points onto the left and right images correspond to non-conjugate points. For example, the image points (b_l_) and (b_r_) in Figure 4 correspond to the object points C and A, respectively. Hence, the grey values at the projections of point B on the left and right warped images will come from different objects on the wall and ground, respectively. Based on the discussion mentioned above, direct comparison of features and spectral information between these images will be possible while eliminating the geometric distortions, which exist in the original imagery. It is for this reason that this integration is referred to as an object-based integration. Moreover, the utilization of warped imagery for feature matching process will take an advantage of both higher planimetric accuracy of the image-based reconstruction and higher vertical accuracy of LiDAR data. Hence, all the following procedures for precise boundary-segment derivation will utilize warped images instead of using the original imagery.

### 3.3. Straight Line Detection

**Figure 5.**
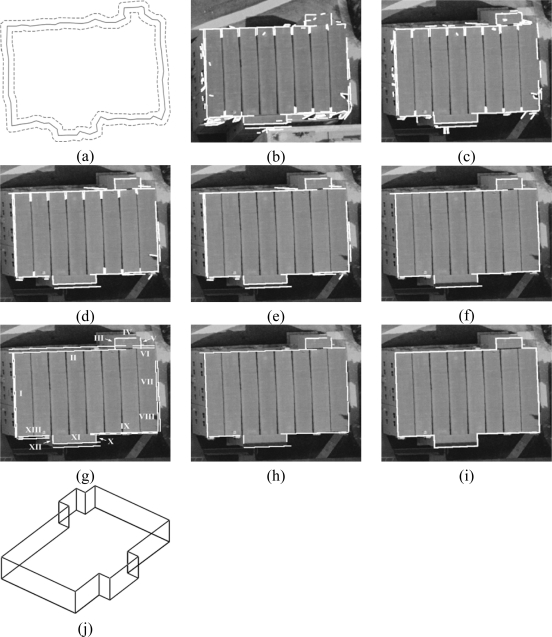
(a) Initial boundary of the building primitive and a buffer surrounding the boundary; straight line segments detected in the (b) left and (c) right warped images; (d) matched 3D lines; (e) filtered matched 3D lines; (f) established reference lines; (g) grouped matched 3D lines; (h) established precise boundary segments; (i) closed-polygon generated from the established precise boundary segments; and (j) generated DBM wire frame.

Now that the concept of warped imagery has been introduced, linear features which are extracted directly from the warped images are utilized in this boundary segment generation method. For each of the building primitives, a buffer surrounding the initial boundary of the building primitive is defined where a straight line detection procedure is applied. The buffer size on each side of the initial boundary is chosen to be approximately equivalent to the average point spacing of the LiDAR points while considering the noise level of the initial boundary. The procedure for linear feature extraction from the warped images encompasses edge detection, edge linking, edge splitting, edge merging, and straight-line fitting. Figure 5(a) shows the initial boundary of the building primitive and the defined buffer. Figure 5b,c shows the straight line segments detected in the left and right warped images, respectively.

### 3.4. 3D Line Matching

**Figure 6.**
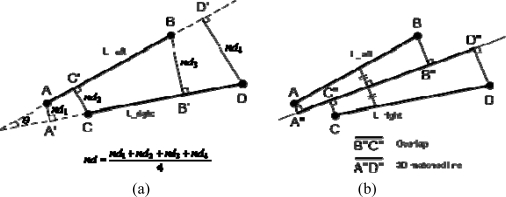
Matching straight line segments in warped images using (a) angle and normal distance constraints and (b) the presence of overlap and 3D matched lines.

The next step involves the matching of straight line segments detected in the left and right warped images. Two line segments (e.g., line segments, AB and CD from the left and right warped images, respectively; see Figure 6) are considered to be matching when the segments satisfy three geometric constraints: angular deviation, normal distance, and presence of overlap. More specifically, we check whether the angle (*θ*) and the normal distance (*nd*) between these segments are smaller than given thresholds (see Figure 6(a)) and whether overlap between the two segments after their projection onto the line bisecting the space between them exists (B″C″ in Figure 6(b)). After this process, each matching pair will define a matched 3D line by determining the extreme points among the projected endpoints along the line bisecting the space between the two line segments in question (the resulting line is A″D″ in Figure 6(b)). Figure 5(d) shows the matched 3D lines for the straight line segments in Figures 5(b) and 5(c).

**Figure 7.**
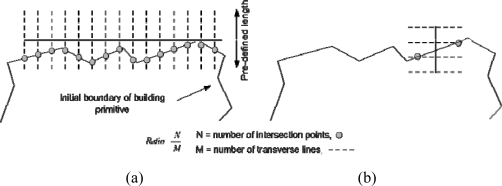
(a) A matched 3D line that follows the trend of the initial boundary of the building primitive and (b) one that does not.

As seen in the figure, some of the matched 3D lines have significantly different orientations than the initial boundary of the building primitive in question. Assuming that the segmentation result is reliable, the initial boundary of the building primitive provides a good overall building shape trend. Hence, the orientation of the matched 3D line under investigation is compared to that of the initial boundary of the building primitive. Then, the matched 3D line is removed if it has significantly different orientation than the initial boundary. More specifically, the transverse lines (i.e., the dashed lines in Figure 7) which are orthogonal to the matched 3D line, with pre-defined lengths (e.g., two times the average point spacing of the LiDAR points to account for potential deviation from the precise building primitives), are spaced at regular intervals along the matched line (i.e., the solid lines in Figure 7).

The intersections of the transverse lines with the initial boundary are derived. The closest intersection points (i.e., circles in Figure 7) to the matched line are identified in this process. Afterwards, the ratio of the number of the intersection points to the number of transverse lines is calculated. For a matched 3D line that follows the trend of the building shape as defined by the initial boundary, this ratio will be close to one (Figure 7(a)). Conversely, the ratio for a line that does not follow the trend will be much less than one (Figure 7(b)). Only matched 3D lines whose ratios are greater than a pre-defined value are kept and utilized in the following procedures.

Figure 5(e) shows the matched 3D lines after filtering out the lines that do not follow the trend of the building shape as represented by the initial boundary of the building primitive. At this stage, one should note that several matched 3D lines competing for the same boundary segment might exist as can be seen in Figure 5(e). Linear features which are closely located and have similar orientations to the initial boundaries of the building primitives (i.e., linear features on the rooftops) might cause this problem. The following subsections will handle this problem by introducing a spectral constraint.

### 3.6. Grouping of Matched 3D Lines

The objective of this step is to divide the filtered matched 3D lines into groups of non-overlapping line segments, which are believed to be competing for the same boundary segment of the building primitive in question. The division is done by establishing a reference line for each of these groups. A matched 3D line will be selected as a reference line if it is the longest line segment among neighbouring/overlapping line segments and is located within the buffer surrounding the initial boundary of the building primitive in question. Once the reference lines are established, the matched line segments will be grouped together based on their angular deviation, proximity, and overlap with the reference lines (i.e., using the same 3D line matching constraints in Figure 6). Finally, all the matched line segments in the same group are extended to the extreme points in that group. Figure 5f,g shows the reference lines and the respective groups, established using the filtered matched 3D lines in Figure 5(e).

### 3.7. Precise Boundary Segment Selection

The aim of this step is to select one line segment, which is believed to represent a precise boundary segment for the building primitive in question, from each of the established groups. A spectral constraint is defined to select the precise building boundary segment among the members of each group. For this constraint, the similarity of the colour values (i.e., a spectral similarity measure) on either side of each of the group members in the left and right warped images is investigated. The precise boundary segment will be defined as the line segment where we have the biggest transition from high to low spectral similarity measures among the line segments in each group. In other words, the line segment which has the largest difference between a high spectral similarity measure on one side and a low spectral similarity measure on the other side will be chosen to be the precise boundary segment.

**Figure 8.**
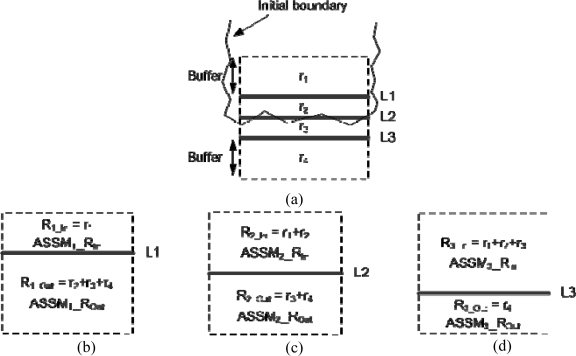
(a) Configuration of the regions around the matched 3D line segments in one group and regions on either side of the line segments (b) L1, (c) L2, and (d) L3.

More specifically, the spectral similarity measure at a given location along the building primitive plane (or warped image plane) is defined as the cosine of the angle between the Red Green Blue (RGB) colour vectors [26] at the same location in the left and right warped images. Regions around the line segments in a particular group are defined (refer to Figure 8(a)). For example, the region r_2_ is bounded by L1 and L2. Also r_3_ is bounded by L2 and L3. Additionally, two more regions, r_1_ and r_4_, are defined by outermost lines and pre-determined buffers (i.e., 2.0 m which is large enough to generate significant similarity measure transitions). Afterwards, each line segment divides the aggregated regions (i.e., r_1_ + r_2_ + r_3_ + r_4_) into two parts. For example, L1 defines two regions, R_1_In_ (= r_1_) and R_1_Out_ (= r_2_ + r_3_ + r_4_), as shown in Figure 8(b). For each line segment, two average spectral similarity values are computed, one for each of the regions, R_i_In_ and R_i_Out_ (for the i^th^ line segment), using Equation 1. Afterward, the differences between the average spectral similarity values for the regions on either side of each line segment are calculated. After comparing the difference values for the line segments in each group, the line segment with the largest difference value will be selected as the precise building boundary segment for the group (Equation 2). In other words, this process will determine the line segment that maximize spectral similarity on one side (i.e., within the region along the building rooftop) and minimize that on the other side (i.e., outside the building rooftop region) as the precise boundary segment. Figure 5(h) shows the precise boundary segments established for the groups in Figure 5(g).

(1) $${\mathrm{ASSM}}_{i}\_R=\frac{\sum_{t=1}^{m}{\mathrm{SSM}}_{t}}{m}$$

(2) $$\mathrm{Selected}\;\mathrm{boundary}\;\mathrm{segment}=\text{arg}\;\underset{\forall i}{\text{max}}\left|{\mathrm{ASSM}}_{i\;}\_R_{\mathrm{In}}-{\mathrm{ASSM}}_{i}\_R_{\mathrm{Out}}\right|,i=1,\ldots n$$

Where *ASSM_i__R* is the average spectral similarity measure for a region on either side of the i^th^ line segment in the group under consideration. *SSM_t_* is the spectral similarity measure for a certain point. *m* is the total number of cells in a certain region along the building primitive plane (or warped image plane). *n* is the total number of line segments in the same group.

**Figure 9.**
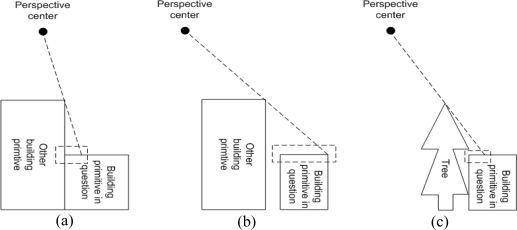
Different types of occlusions: caused by (a) a higher building primitive sharing a vertical wall with the one in question; (b) a higher and non-adjacent building primitive; and (c) trees closely located to the primitive in question.

One should note that some of the building boundary portions might not have precise boundary segments from the integration process due to the failure of 3D line matching process. Such a failure comes from the occlusions where other higher building primitives or trees might hide the building primitive in question either partially or fully. Figure 9a–c illustrates different types of occlusions (i.e., areas enclosed by the dashed lines in the figures) caused by a higher building primitive sharing a vertical wall with the one in question, a higher and non-adjacent building primitive, and trees closely located to the building primitive in question, respectively.

**Figure 10.**
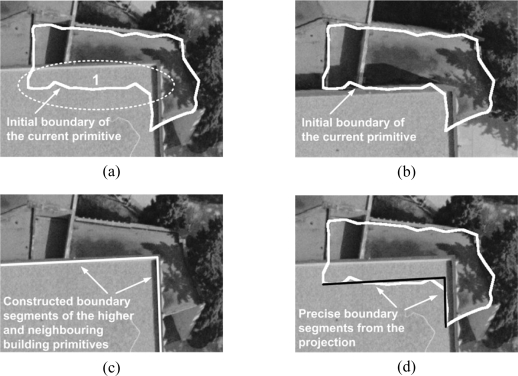
Relief displacement from higher and neighbouring building primitive in (a) left and (b) right warped images; (c) Constructed boundary segments of the higher and neighbouring building primitive; and (d) precise boundary segments for the current primitive after projection process and trimming.

Figure 10a,b shows a real example of the first type of occlusion. As can be seen in the figures, the initial boundary after its projection onto the left and right warped images is very close to the boundary segments of the building primitive in question except the portions within area 1, which are enclosed by dashed ellipse. The portion of the projected boundary in area 1 seems to be far from the observed building boundaries in the left warped image in Figure 10(a). Such a deviation is the result of relief displacement from the higher and neighbouring building primitive and leads to the failure of 3D line matching in the area 1. In other words, the observed image boundaries in area 1 do not correspond to the boundaries of the building primitive in question. Rather, they belong to the boundaries of the higher neighbouring primitive. To resolve this problem, all the initial boundary points that are closely located to the derived boundary segments from the integration process are first identified and removed from the boundary in question. Then, the precise boundary segments corresponding to the remaining boundary points will be derived through the following process while considering different types of occlusions.

## 4. Precise Boundary Segment Derivation through Hierarchical Processing of Building Primitives

The remaining portions of the initial boundary corresponding to the occlusion areas caused by higher building primitives sharing vertical walls with the ones in question (see Figure 9(a)) can be precisely reconstructed through the hierarchical processing of building primitives. The constructed precise segments of the higher and neighbouring building primitives will be utilized for the lower building primitive. More specifically, the solution for the lower building primitives that share vertical walls with higher building primitives can be derived from the projection of the constructed precise segments of higher building primitives onto lower, neighbouring building primitives, if any exists. To do that, a 2D adjacency table that identifies neighbouring building primitives should be defined after the projection of their initial boundaries onto a horizontal plane. In the adjacency table, two building primitives will be deemed as neighbours if part of the initial boundary of one primitive is located within a buffer zone surrounding the boundaries of the other primitive. Figure 10c,d shows a real example for the boundary segment generation based on the projection. The constructed boundary segments of the higher and neighbouring building primitive are shown as white solid lines in Figure 10(c). These lines are projected onto the current primitive and their end points are redefined by checking the proximity between the projected lines and the points of the initial boundary of the current primitive. Black solid lines in Figure 10(d) indicate boundary segments for the lower primitive which are obtained after the projection process and trimming. Then, all the initial boundary points that are closely located to the derived boundary segments from the hierarchical processing are identified and removed from the boundary in question.

At this stage, one should note that there might be portions of the initial boundaries which have not been refined. This might happen when higher building primitives or trees partially or fully hide lower and non-adjacent building primitives in the imagery (see Figure 9b,c). The right side of the building primitive in Figure 10 also shows trees partially hiding the building primitive in question. The shadow effects and low image contrast might affect the edge detection process leaving some gaps in the defined precise boundaries. To resolve this problem, we will utilize only the initial boundary portions to define a refined boundary. More specifically, the building-primitive boundary will be reconstructed by regularizing the remaining initial boundaries of the building primitives using the Douglas-Peuker method [27] and a straight-line fitting algorithms through a least squares adjustment.

## 5. Closed Polygon and 3D Building Model Generation

So far, precise boundary segments have been established through the procedures mentioned above. One should note that the established segments are not connected to each other. This section will, hence, introduce a procedure to make a closed polygon using these segments. This procedure begins by establishing the proper sequence of the established boundary segments for each building primitive, by investigating their proximity to the ordered chain of vertices along the initial boundary for that primitive. To construct a closed-polygon for a given building primitive, an intersection procedure is implemented to connect neighbouring boundary segments according to their established sequence. Figure 5(i) shows the closed-polygon generated from the previously established boundary segments in Figure 5(h).

**Figure 11.**
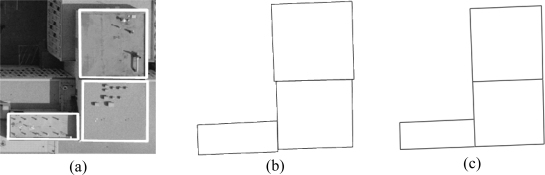
(a) Closed-polygons for three building primitives at different elevations; (b) boundary segments projected onto a horizontal plane; and (c) co-aligned boundary segments.

The boundary refinement and closed-polygon generation procedures are carried out for the individual building primitives one by one starting from the highest building primitives and proceeding to lower ones. Figure 11(a) shows a real example for the polygons defined for three building primitives, which are located at three different elevations. Moreover, the boundary segments projected onto a horizontal plane are shown in Figure 11(b). As can be seen in the figure, there might be misalignments between the established boundaries for neighbouring building primitives at different elevations. Hence, the last step in the DBM generation process is the automated simultaneous co-alignment of the boundary segments for all the building primitives. The alignment process starts by identifying neighbouring building primitives through a 2D adjacency analysis in the same manner mentioned in Section 4. Then, for the neighbouring building primitives, one investigates the proximity and the degree of parallelism of the projected boundary segments onto the horizontal plane. Boundary segments that meet pre-specified thresholds, which define the acceptable range of collinearity/parallelism, normal distance, and distance between the end points for two neighbouring boundary segments, will be merged through a straight-line fitting procedure. Finally, the merged lines are projected back onto their respective building primitive planes. Figure 11(c) shows the co-aligned boundaries after the alignment process. After the boundary alignment procedure has been carried out, a DBM wire frame is generated by connecting the vertices of the co-aligned boundary segments along the building rooftops with other versions of these boundary vertices at the terrain elevation, which is defined as the average elevation of the neighbouring terrain points (i.e., the buildings’ footprints). Figure 5(j) shows a DBM wire frame generated using the polygon in Figure 5(i).

**Figure 12.**
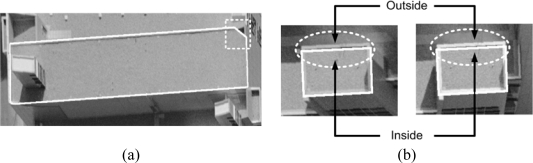
Erroneous boundary segments produced through the refinement process due to (a) shadow and (b) a weak similarity transition in two warped images.

The automatically generated DBM might include incorrectly detected boundary segments. Two examples of erroneous boundary segments are shown in Figure 12. The line segment in the area bounded by the white box in Figure 12(a) is produced by the edges of the shadow around the fence on the rooftop. Since the shadow is close to the initial boundary of the building primitive, the probability that the shadow line will be detected as a boundary segment is relatively high. On the other hand, Figure 12(b) shows a different type of erroneous boundary segment. The regions on either side of the line segment in question are almost homogeneous in the two warped images (check the line segments and surrounding region enclosed by the white dashed ellipse). Edge lines which are parallel to the base line (i.e., a line connecting two perspective centers) and with uniform texture on both sides might have low similarity difference from one side of the line segments to the other. Eventually, this weak transition might lead to wrong selection of the precise boundary segment.

To edit incorrectly established boundary segments from the automated process, a manual mono-plotting procedure is introduced. The operator interactively deletes or adds boundary segments to acquire complete building models. More specifically, the measurements of the end points for the erroneous boundary segments are done in one image and a mono-plotting procedure is used to directly project these measurements onto the plane of building primitives using the established geo-referencing parameters. It should be noted that the operator does not need to precisely measure the end points of the boundary segments (i.e., the operator needs to just make sure that the measured points lie along the boundary segments). The end points of the boundary segments will be precisely defined through the automated closed-polygon generation procedure.

## 6. Experiments and Evaluations

**Figure 13.**
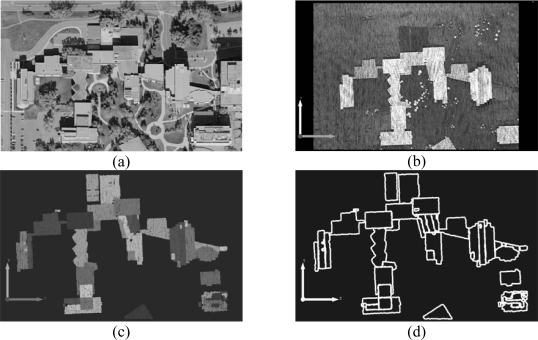
(a) Aerial photo over the area of interest; (b) LiDAR points over the same area; (c) clustered building primitives; and (d) the initial boundaries of the building primitives.

Experiments with real LiDAR and image data are carried out to evaluate the performance of the proposed methodology. The LiDAR dataset and a stereo-pair were acquired by an Optech 3100 system and RC30 analog camera, respectively. The stereo-pair has a scale of 1:5,000 and was digitally scanned at a resolution of 12 microns, resulting in a Ground Sampling Distance (GSD) of 6 cm. The Interior Orientation Parameters (IOPs) were provided in a Camera Calibration Certificate (CCC) and the Exterior Orientation Parameters (EOPs) were determined through the proposed co-registration procedures in Section 3. The average point spacing for the LiDAR data is approximately 0.75 m (about 1.3 points per square meter). Figure 13a,b shows a portion of the image and LiDAR data gathered in the area of interest. As shown in Figure 13(a), this area has complex and connected buildings, as well as some trees and mild terrain variation. The LiDAR points in Figure 13(b) are assigned different shades of grey according to their heights. Through the building hypothesis and primitive generation procedures, we achieved the building primitives in Figure 13(c) and their initial boundaries in Figure 13(d).

In the next processing phase, the initial boundaries of the building primitives are refined through the precise boundary segment derivation. The precise boundary segments are derived from the integration of LiDAR and photogrammetric data and hierarchical processing of the building primitives. Once all the boundary segments for a building primitive have been generated, a closed-polygon is generated using these boundary segments. Finally, all the boundary segments of the closed-polygons in the study area are aligned simultaneously. The descriptions, values, and justifications for the utilized thresholds in the precise boundary segment derivation are listed in Table 1.

**Figure 14.**
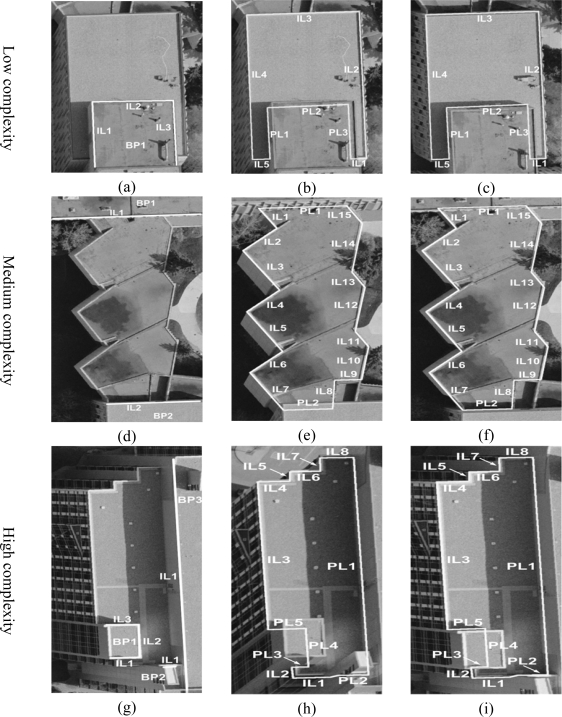
Building primitives with different complexity levels: (a, d, and g) derived line segments for the higher and neighbouring building primitives and refined boundaries projected onto the (b, e, and h) left and (c, f, and i) right warped images.

**Figure 15.**
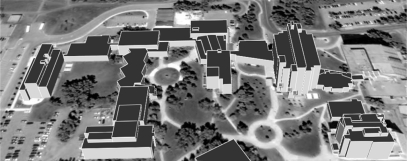
DBM (in KML format) produced through the automated building reconstruction process is imported into Google Earth.

The performance of the proposed precise boundary segment derivation procedures can be verified by visually checking the closeness of the automatically produced boundaries to the actual building primitive boundaries. Three building primitives are selected to assess the performance of the boundary refinement procedure for different levels of building complexity. Figures 14 shows the refined boundaries for these building primitives projected onto the left and right warped images. Figure 14a–c, 14d–f, and 14g–i shows building primitives with low, medium, and high complexity, respectively. Also, Figure 14a,d,g shows the line segments belonging to the higher and neighbouring building primitives. The boundary segments derived through the integration and projection processes are denoted as IL and PL, respectively. For a building primitive with low complexity in Figure 14a–c, five line segments (IL1–IL5) are extracted through the integration of the imagery and LiDAR data. In addition, three line segments (PL1–PL3) are derived by projecting the boundary segments (IL1–IL3) of a higher and neighbouring building primitive (i.e., BP1) onto the building primitive in question. In the same manner, the building primitives with medium and high complexity are reconstructed in Figure 14d–f and 14g–i, respectively. The visual inspection proved that the proposed building reconstruction procedure performs well for complex and connected buildings. As a final product of the automated building reconstruction process, the DBM is generated and converted to KML format, which can be directly imported to Google Earth, as shown in Figure 15.

This part of the experimental results provides a quantitative evaluation of the correctness, completeness, and accuracy of the proposed automated DBM generation process. The correctness and completeness measures, Equations (3) and (4), respectively, introduced by [28] are adopted for this evaluation.

(3) $$\mathrm{Correctness}=\frac{\mathrm{Number}\;\mathrm{of}\;\mathrm{correctly}\;\mathrm{determined}\;\mathrm{boundary}\;\mathrm{segments}}{\mathrm{Total}\;\mathrm{number}\;\mathrm{of}\;\mathrm{established}\;\mathrm{boundary}\;\mathrm{segments}}$$

(4) $$\mathrm{Completeness}=\frac{\mathrm{Number}\;\mathrm{of}\;\mathrm{correctly}\;\mathrm{determined}\;\mathrm{boundary}\;\mathrm{segments}}{\mathrm{Total}\;\mathrm{number}\;\mathrm{of}\;\mathrm{actual}\;\mathrm{boundary}\;\mathrm{segments}}$$

The correctness measure evaluates the percentage of erroneous boundary segments among the established boundary segments. On the other hand, the completeness measure gives an indication of the percentage of missing or erroneously established boundary segments. The study area has a total of 40 building primitives, which are represented by 291 boundary segments. The proposed procedure produced 311 boundary segments for all the building primitives. Among the boundary segments produced, 276 boundary segments were correctly established. Based on these numbers, the correctness and completeness measures for this study area are 89% (276/311) and 95% (276/291), respectively. Several researchers have used the correctness and completeness measures to evaluate the quality of their DBM reconstruction. The correctness of 80% and the completeness of 94% in a per-pixel evaluation were achieved by [29]. 81% of buildings (in completeness) in a per-building evaluation were detected by [19]. Moreover, buildings at the rate of 90.1% in correctness and 88.3% in completeness were detected by [30] based on a per-pixel evaluation. One should note that it is difficult to directly compare the performances of different building reconstruction methodologies since they utilized different datasets with different complexity. However, in general, we can conclude that the proposed building reconstruction approach provides slightly higher performance compared to other researches. Moreover, this research utilized a simple manual mono-plotting procedure to edit incorrectly established boundary segments from the automated process and to acquire complete building models.

As for assessing the accuracy of the automatically established DBM, the coordinates of the DBM corner points are compared with those derived manually using a photogrammetric reconstruction procedure. To get an idea of the quality of the manually derived coordinates, two operators were asked to derive the 3D coordinates of the vertices of the building rooftops in the study area. The second column in Table 2 shows the mean, standard deviation, and Root Mean Squared Error (RMSE) values for the differences between the derived coordinates by each of the two operators. Based on the mean values, one can see that there are no systematic discrepancies between the coordinates derived by the two operators. The planimetric RMSE values are in the range of six pixels, which is mainly due to the difficulty of accurately pinpointing the corner points in the imagery.

**Figure 16.**
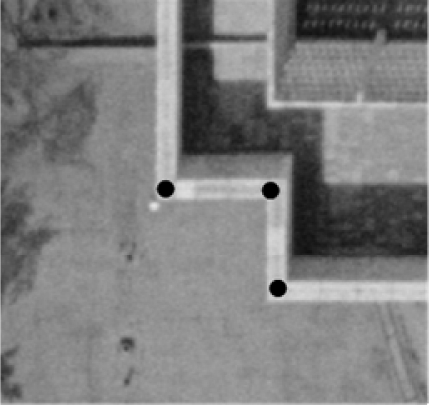
A building with protruding fences with manually selected corner points.

Having established the expected accuracy from a manual operation, the coordinates derived from the proposed procedure were compared with the average of the coordinates determined by the two operators. The statistics derived from this comparison are reported in the third column of Table 2. The reported planimetric mean, standard deviation, and RMSE values in the third column are quite close to the results provided by the two operators, which indicates very high accuracy of the derived coordinates. The mean and RMSE values in the Z-direction, on the other hand, seem to be much worse. The reason for this deterioration is that most of the buildings in the study area had fences on their rooftops, as seen in Figure 16.

The heights of these fences range from 0.5 m to 1.0 m. The operators defined the corners of the DBM on top of the fences (the figure shows the points selected by the operators), whereas the proposed procedure placed the corners on the rooftop plane, as defined by the segmented LiDAR data. Therefore, the mean comparison shows a Z-bias between the manually and automatically generated vertices (of almost 60 cm, which is an indication of the average heights of the fences). The standard deviation in the Z-direction reveals good compatibility between the manual and automated procedures.

## 7. Conclusions and Recommendation for Future Work

This research proposed a new methodology for automated generation of polyhedral building models for complex structures with horizontal and tilted rooftops, which are bounded by straight lines. The key contributions of this research in the building reconstruction procedure are the precise boundary segment derivations from two processes, namely, one through the integration of photogrammetric and LiDAR data; and the other one through the hierarchical processing of building primitives.

An object-based integration of photogrammetric and LiDAR data has been carried out in one-step by introducing warped imagery without handling photogrammetric and LiDAR data separately. The image matching ambiguity was significantly reduced by utilizing warped imagery since such imagery does not have relief displacement and geometric distortions. Moreover, the utilization of warped imagery for feature matching process took an advantage of both higher planimetric accuracy of the image-based reconstruction and higher vertical accuracy of LiDAR data. In addition, spectral similarity transitions across the matched line segments in the warped imagery were investigated to select precise boundary segments. The hierarchical processing of building primitives was proposed to reconstruct boundary segments in occluded areas where higher building primitives hide lower and neighbouring primitives. Precise boundary segments have been acquired for such occluded areas by projecting the boundary segments constructed for higher building primitives onto the lower and neighbouring ones.

Qualitative analysis was performed by visually inspecting the constructed boundary results. The visual inspection proved that the proposed building reconstruction procedure performs well for complex and connected buildings. Quantitative analysis was also carried out based on the completeness, correctness, and RMSE analysis. The correctness and completeness ratios for the study area were 89% and 95%, respectively. The performance of this research is slightly higher compared to other studies. Missing or erroneous line segments can be edited through an introduced simple manual mono-plotting work; then, complete building models can be easily acquired. In addition, the coordinates of the DBM corner points were compared with those derived manually using a photogrammetric reconstruction procedure to assess the accuracy of the established DBM. The RMSE analysis found that the DBM produced was quite close to the results obtained manually.

Future work will focus on the alleviation of the effects of shadow and uniform textures on both sides of a boundary segment. Moreover, the building reconstruction procedure will be expanded to incorporate more than two images. Finally, this approach will be extended to manipulate non-planar surfaces, which might not be bounded by straight lines.

## Figures and Tables

**Table 1.** Descriptions, values, and justifications for the utilized thresholds in the precise boundary segment derivation.

**Threshold**	**Description**	**Value**	**Justification**
Edge detection buffer	Size of the buffer on each side of the initial boundary used for edge detection	1.0 m	Approximately equivalent to the average point spacing of the LiDAR data (on each side of the initial boundary)
Matching angle	Acceptable angle between candidate line segments in the matching process	6 degrees	Based on the accuracy of the geo-referencing parameters and the noise level in the detected edges
Matching normal distance	Acceptable normal distance between candidate line segments in the matching process	0.5 m	Based on the accuracy of the geo-referencing parameters and the noise level in the detected edges
Ratio for filtering 3D matched lines	Utilized ratio to exclude 3D matched lines that do not follow the trend of the initial boundary	0.6	Based on the noise level in the defined initial boundary
Angle threshold for grouping 3D matched lines	Angle threshold for grouping 3D matched lines, which are believed to compete for the same building boundary	6 degrees	Based on the accuracy of the geo-referencing parameters and the noise level in the detected edges
Normal distance threshold for grouping 3D matched lines	Normal distance threshold for grouping 3D matched lines, which are believed to compete for the same building boundary	1.0 m	Approximately equal to the edge detection buffer size
Buffer for outmost regions	Buffer size for reliable spectral similarity measure derivation within the precise boundary segment selection process	2.0 m	Large enough to generate a representative spectral similarity measure

**Table 2.** Statistics derived from the comparison of two manually generated sets of DBM vertices (second column) as well as the comparison of automatically and manually generated DBM vertices (third column).

	**Manual DBM**	**Automated DBM**
No. of vertices	116	78
Mean (X), m	−0.086	−0.040
Mean (Y), m	−0.008	0.003
Mean (Z), m	−0.091	0.553
Std_dev (X), m	±0.349	±0.392
Std_dev (Y), m	±0.364	±0.407
Std_dev (Z), m	±0.239	±0.237
RMSE (X), m	0.357	0.392
RMSE (Y), m	0.362	0.405
RMSE (Z), m	0.255	0.601
